# Putting the Polio Workforce to Work in a Public Health Crisis: Contributions of the National Stop Transmission of Polio (NSTOP) Program to the COVID-19 Response in Pakistan

**DOI:** 10.3390/vaccines13080875

**Published:** 2025-08-19

**Authors:** Aslam Pervaiz, Rana Muhammad Safdar, Mumtaz Ali Laghari, Nadeem Shah, Amjad Mehmood, Kifayat Ullah, Richard Franka, Chukwuma Mbaeyi

**Affiliations:** 1 National Stop Transmission of Polio (NSTOP) Program, Islamabad 44000, Pakistan; 2 Pakistan National Emergency Operations Center (NEOC) for Polio Eradication, Islamabad 44000, Pakistan; 3 Global Immunization Division, Global Health Center, Centers for Disease Control and Prevention, Atlanta, GA 30329, USA

**Keywords:** COVID-19, pandemic, outbreak response, Pakistan, polio, vaccination

## Abstract

**Background**: Pakistan reported its first case of COVID-19 in February 2020 and joined other countries in activating a national emergency response following the declaration of the COVID-19 pandemic by the World Health Organization (WHO). Playing a vital role in the early phase of the country’s response was the National Stop Transmission of Polio (NSTOP) program, a highly trained cadre of polio workers who ordinarily support polio eradication efforts in the country. **Methods**: We developed a reporting tool using Microsoft Excel that tracked the activities of NSTOP officers to support the COVID-19 response. All NSTOP officers submitted their activity reports fortnightly using this reporting tool. Each provincial NSTOP officer reviewed and compiled their respective officers’ reports and sent them to the federal NSTOP Team. We present a summary of the reports for the period from 1 March 2020 to 31 July 2020. **Results**: A total of 71 officers of the NSTOP program supported various aspects of Pakistan’s COVID-19 response, including coordination, detection and response activities, surveillance, quarantine/isolation management, training and orientation sessions for healthcare personnel, data analysis, community engagement, and risk communication. They successfully investigated 32,729 suspected COVID-19 cases, of which about one-third were confirmed cases, and facilitated the collection and dispatch of >57,000 samples from these cases. **Conclusions**: This report details NSTOP contributions to the early phase of the COVID-19 response in Pakistan, demonstrating the value of polio investments beyond eradicating the disease to encompass having a workforce that is ready to respond to emergent disease threats and outbreaks. Such a workforce could also play a role in strengthening the capacity of existing immunization systems to help improve routine vaccination coverage in resource-limited settings.

## 1. Background

Twenty-seven patients with pneumonia of unknown etiology were identified in Wuhan City, Hubei province, China, on 31 December 2019 [1,2]. On 7 January 2020, throat swab samples from these patients detected the causative agent as a novel coronavirus, which was then named Severe Acute Respiratory Syndrome Coronavirus 2 (SARS-CoV-2). The World Health Organization (WHO) designated the accompanying clinical disease caused by the virus as Coronavirus Disease (COVID-19) [3]. By 30 January 2020, WHO had declared the COVID-19 outbreak a public health emergency, with a subsequent declaration of the disease as a global pandemic on 11 March 2020 [4,5]. The WHO emergency committee advised that the spread of COVID-19 could be interrupted by early case detection, isolation, prompt treatment, and the implementation of a robust system to trace contacts [6].

Pakistan reported its first case of COVID-19 on 26 February 2020 [7]. The Government of Pakistan and international partner organizations working in the country responded promptly to the COVID-19 public health emergency. One of such key partners to the Government of Pakistan is the Global Polio Eradication Initiative (GPEI), an umbrella organization overseeing polio eradication activities in partnership with ministries of health around the world. GPEI is led by WHO, UNICEF, Rotary International, the United States Centers for Disease Control and Prevention (CDC), and the Bill & Melinda Gates Foundation [8]. The partnership supports the Ministry of Health in Pakistan in implementing poliovirus surveillance and immunization activities based on a set of global guidelines. Following the onset of the COVID-19 pandemic, GPEI suspended the implementation of polio vaccination campaigns in Pakistan until June 2020, and all polio staff were rerouted to support COVID-19 response activities beginning in March 2020. On the directive of the Federal Ministry of National Health Services Regulations and Coordination, and with the endorsement of the National Polio Management Team, the Pakistan Polio Eradication Initiative (PEI) team reorganized and realigned its roles and responsibilities as per emerging needs at all levels including the National Emergency Operations Center (NEOC), Provincial Emergency Operations Centers (PEOCs), and at the district level to fully support the COVID-19 response.

A contribution of particular value to the COVID-19 response in Pakistan was the deployment of National Stop Transmission of Polio (NSTOP) officers to support the response in key provinces across the country. The NSTOP program was established in March 2011 as a collaborative initiative of the Field Epidemiology and Laboratory Training Program (FELTP) of the Ministry of Health (MoH), the Government of Pakistan, the National Expanded Program on Immunization (EPI), Provincial Health Departments, WHO, and CDC to strengthen polio eradication activities in Pakistan, where poliovirus circulation remains endemic [9]. The NSTOP program, modeled after the global STOP program, recruits and trains medical and public health professionals, including graduates of FELTP, to support polio surveillance and immunization activities at the district level in three provinces, Balochistan, Sindh, and Khyber Pakhtunkhwa, as well as in Islamabad. From an initial cohort of 16 NSTOP assignees, the program grew to 81 NSTOP officers in 2019, working in 61 districts designated by the polio program as high-risk.

With the onset of the COVID-19 pandemic, NSTOP officers were assigned to support the national, provincial, and district EOCs with developing and implementing a strategic response to the outbreak in Pakistan. NSTOP officers had been instrumental in the establishment of district EOCs for polio eradication activities and were tasked with utilizing the same system to support the country’s COVID-19 response. This article highlights the contributions of the NSTOP program to the COVID-19 pandemic response in Pakistan during the early phase of the response from 1 March 2020 to 31 July 2020.

### 1.1. NSTOP Program Description

The NSTOP program is designed to train a team of local public health professionals on the essentials of polio eradication and improving routine immunization and then deploying them to high-risk districts as designated by the polio program. Medical professionals with at least two years of public health experience are recruited from among public sector doctors who were either alumni/residents of the two-year FELTP or had undergone four weeks of specialized training in surveillance and outbreak detection and response conducted by FELTP Pakistan. Selection criteria include the following: (1) Candidates’ residence within or close to the area where they will work, as they are expected to be knowledgeable of the culture and local issues of the communities they serve; (2) enrollment on the payroll of the respective provincial health departments; (3) work experience in polio eradication or the Expanded Program on Immunization; and (4) language proficiency and appropriate technical competencies. Typical assignments are for a period of one year with performance-based extensions. Long-term deployments are discouraged in security-compromised areas where polio workers have been targeted.

Pre-deployment training is based on the global STOP program curriculum with adaptations to fit the local context. Training sessions are conducted over a two-week period and include instruction and case studies on (1) poliovirus epidemiology and fundamentals of polio eradication strategies; (2) National Emergency Action Plan (NEAP) components relevant for the role of NSTOP officers in implementing polio eradication activities within an emergency response; (3) vaccination campaign planning, implementation, monitoring and evaluation; (4) acute flaccid paralysis (AFP) surveillance; and (5) strengthening routine immunization. During quarterly review meetings, refresher training sessions are conducted over a three- to five-day period.

NSTOP officers are assigned to district emergency operations centers (DEOCs), as well as provincial and federal EOCs, to work on all polio eradication activities, including the planning, implementation, and monitoring of vaccination campaign activities, strengthening of AFP surveillance, investigation of polio cases, and outbreak response. They also support coordination among the local health department, district administration officials, and international partners and in tracking the implementation of the NEAP. The supervision of officers is through routine calls, weekly and monthly written reports, and regular site visits by the federal NSTOP team.

### 1.2. NSTOP Officer Participation in COVID-19 Response

**Figure 1:**
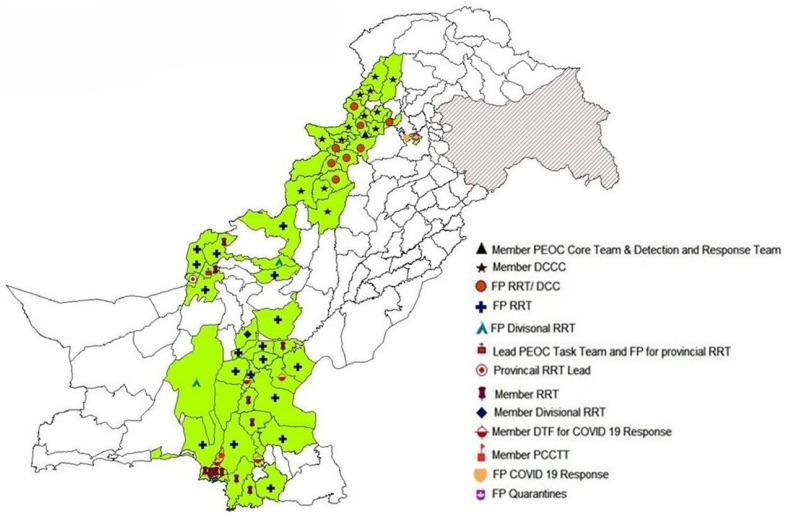
NSTOP officer roles and responsibilities in COVID-19 response, Pakistan, March–July 2020. Abbreviations: DCCC = District Command and Control Committee; FP = Focal Person; DCC = District Coordination Committee; DTF = District Task Force; PCCC = Provincial Command and Control Committee; RRT = Rapid Response Team; TL = Team Lead; PCCTT = Provincial Command and Control Task Team. The districts where NSTOP officers were assigned are colored green.

**Figure 2:**
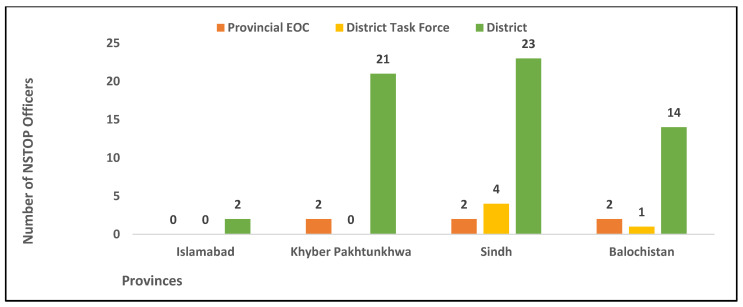
Number of NSTOP Officers deployed by province and within province placement in March–July 2020. Abbreviation: Provincial EOC = Provincial Emergency Operation Center.

With the declaration of the COVID-19 outbreak in Pakistan and on the instruction of the leadership of the NEOC, NSTOP officers, along with other GPEI partner organizations, were engaged in COVID-19 preparedness and response activities at the district, provincial, and national levels. A total of 60 NSTOP officers in 58 high-risk districts, 6 officers in provincial EOCs, and 5 officers within divisional task force (DTF) teams across Pakistan supported the COVID-19 response (Figure 1 and Figure 2). Except for polio vaccination campaigns, the NSTOP team was supporting activities for both polio eradication and COVID-19 response.

## 2. Methods

We developed a reporting tool using Microsoft Excel that tracked the activities of NSTOP officers to support the COVID-19 response. The tool captured 52 activities grouped by the following thematic areas: coordination; case detection and management; risk communication and community engagement; training and orientation sessions; surveillance and screening; data and risk analysis; infection prevention and control; establishment and maintenance of quarantine and isolation stations; and implementation of lockdowns.

All NSTOP officers submitted their activity reports fortnightly using this reporting tool through their respective provincial NSTOP Team Leads (TLs) and Technical Support Officers (TSOs). Each provincial NSTOP office reviewed and compiled its respective officers’ reports and sent them to the federal NSTOP Team. The activity reports were compiled by province and by staff categories (district NSTOP officers, TLs, and Divisional Task Force officers [DTF]). We present a summary of the reports for the period from 1 March 2020 to 31 July 2020. The analysis was conducted using Epi Info version 7.1.5.0.

## 3. Results

### 3.1. Response Coordination

NSTOP officers played a vital role in the COVID-19 Response Coordination Committees at all levels. Provinces had different coordination and command committee designations, namely Provincial Command and Control Committees (PCCCs), District Command and Control Committees (DCCCs), Task Teams, and Rapid Response Teams (RRTs). The NSTOP Team Lead in Balochistan led the Provincial EOC Task Team, while the NSTOP TL in Sindh was a core member of the COVID-19 Task Team. The NSTOP TL for Khyber Pakhtunkhwa was a member of the Provincial Detection and Response Team. The District Task Force NSTOP officers were leads or members of their respective divisional RRTs. Thirty district NSTOP officers were focal persons of district DCCC and RRTs, and twenty-five were members of these committees in their respective districts. Coordination committees met frequently for decision-making and to assess the status of implementation of critical decisions made by the committees. TLs/DTFs facilitated 387 PCCC/Task Team/RRT meetings, and district NSTOP officers facilitated 1717 DCCC/Task Team/RRT meetings. TLs/DTFs also facilitated response-related evening review and daily meetings (TLs/DTFs: 425 and district NSTOP officers: 3358) and attended a total of 450 security meetings (Table 1 shows activities by district officers, by TL/DTF officers, and overall).

### 3.2. Detection and Management of COVID-19 Cases

Provincial NSTOP TLs and NSTOP DTF officers performed detection and response activities in their respective provinces, and district NSTOP officers in their respective jurisdictions. Overall, NSTOP officers investigated 32,729 suspected cases of COVID-19 (average 511 per officer) and 11,503 confirmed COVID-19 cases (average 180 per officer). Real-time reverse transcriptase polymerase chain reaction (RT-PCR) testing was used as a confirmatory test. Additionally, they identified 708 suspected COVID-19 refusals, i.e., those who denied their suspected infection status, and convinced them to accept testing and management according to the results. Table 1 reflects the total numbers accomplished by NSTOP officers at the provincial and district levels and average numbers by officer.

To support COVID-19 case management, NSTOP officers followed 13,713 confirmed cases through home visits/phone calls for up to 14 days; identified and traced 15,283 contacts of COVID-19-positive cases; and coordinated the transfer of 3160 COVID-19-positive cases to isolation centers and health department units (HDUs). They also facilitated laboratory sample collection from 57,193 suspected COVID-19 cases and organized the transport of 54,712 samples to provincial and national laboratories according to protocol (Table 1).

NSTOP officers also supported the compilation of COVID-19 case data that was submitted through WhatsApp or email from the district to provincial and national levels and entry into the Integrated Disease Information Management System (IDIM). They also facilitated detailed data collection of COVID-19-related deaths.

### 3.3. Risk Communication and Community Engagement

**Figure 3:**
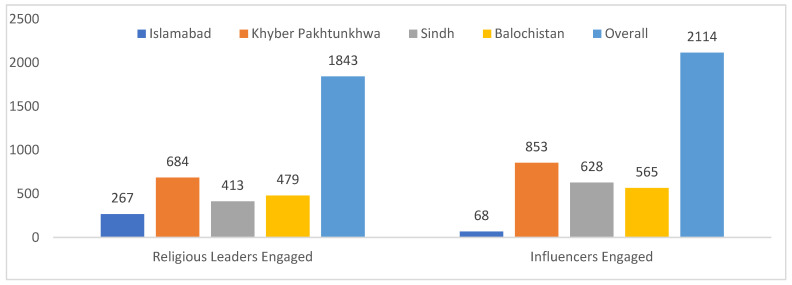
Number of influencers and religious leaders engaged by NSTOP officers during COVID-19 response, by province, Pakistan, March–July 2020.

The NSTOP program supported COVID-19 risk communication through various community engagement activities. NSTOP officers served as technical experts on 234 national and local media (TV/radio) programs to raise awareness of the risk of COVID-19 and how people could protect themselves from it. Further, NSTOP TLs/DTFs officers served 180 times as the technical focal person to the PEOC Coordinator/Divisional Commissioner, while district NSTOP officers served 1126 times as technical representatives for the Divisional Commissioner in interactions with mass media (TV/radio/print) and on social media regarding COVID-19. NSTOP officers also participated in community sensitization sessions and meetings with local religious and civil influencers to increase awareness about COVID-19 risks and recommendations to reduce community transmission and facilitate reporting, treatment, and follow-up of cases and contacts. Overall, NSTOP officers facilitated 1096 community engagement sessions and sensitized 18,434 participants. They also engaged 1897 religious leaders and 2311 influencers. Finally, in the capacity of technical focal persons, NSTOP officers responded to 27,361 public calls to seek advice and guidance about COVID-19 at the divisional and district-level call centers (Table 1, Figure 3).

### 3.4. Training and Orientation Sessions

**Figure 4:**
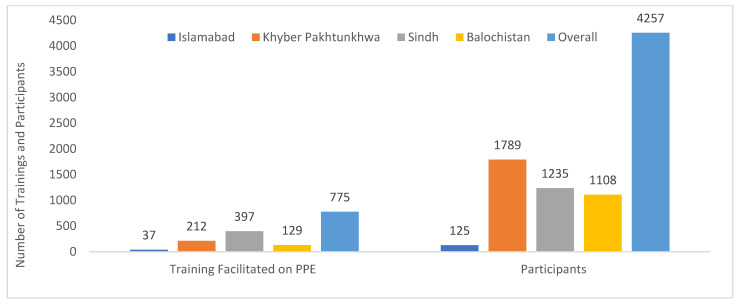
Number of training sessions and attendants to training on the use of personal protective equipment (PPE) facilitated by district NSTOP officers during the COVID-19 response by province, Pakistan, March–July 2020.

NSTOP officers facilitated training and orientation sessions for healthcare providers (HCPs) at the provincial, divisional, and district levels. NSTOP officers facilitated a total of 1204 training sessions on COVID-19 response, with the participation of 1103 HCPs at the divisional level and 8801 HCPs of health departments at the district level (Table 1). NSTOP officers also conducted 828 training sessions on procedures for donning and doffing personal protective equipment (PPE) for 4811 HCPs and monitored 596 training sessions conducted by district public health officials (Table 1, Figure 4).

### 3.5. COVID-19 Surveillance Activities

**Figure 5:**
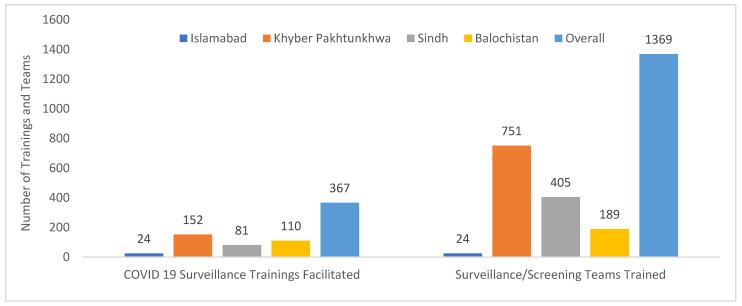
COVID-19 surveillance training sessions facilitated by district NSTOP officers and the number of teams trained by province, Pakistan, March–July 2020.

NSTOP officers are doctors with expertise in disease surveillance based on their FELTP training and work experience in polio surveillance. Because of their technical capacity, they were selected to participate in COVID-19 Surveillance Committee meetings that made decisions on local response and to train field staff involved in disease surveillance and screening. As such, they attended 746 Surveillance Committee meetings, facilitated 417 surveillance training sessions, and trained a total of 1554 surveillance/screening teams (Table 1, Figure 5).

### 3.6. Establishment and Maintenance of Quarantine and Isolation Facilities

**Figure 6:**
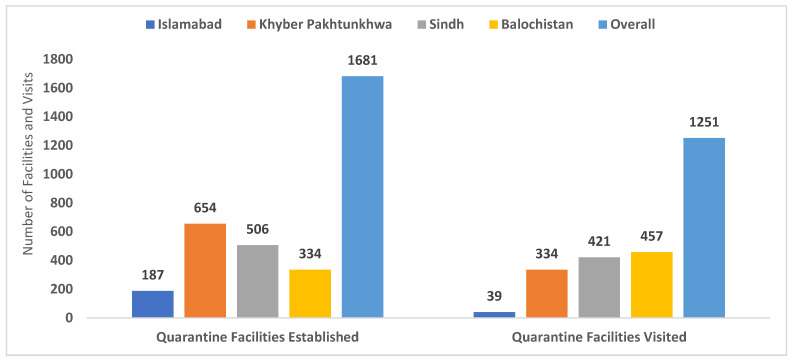
Number of quarantine/isolation facilities established and managed by NSTOP officers during the COVID-19 response, by province, Pakistan, March–July 2020.

NSTOP officers provided technical guidance for the establishment and maintenance of quarantine and isolation facilities at the provincial, divisional, and district levels. NSTOP TLs/DTF officers and district NSTOP officers facilitated the establishment of 1760 quarantine/isolation facilities at their assigned areas of work. NSTOP officers also played a pivotal role in the maintenance and running of these facilities as per standard SOPs. NSTOP officers visited these facilities 1431 times during the study period (March–July 2020) to ensure that the following activities were being implemented: real-time risk assessment of quarantine camps; maintenance of a clean workspace at the COVID-19 control room on site; availability and proper use of PPE; appropriate waste management; and disinfection of vacated rooms before reassignment (Table 1, Figure 6).

### 3.7. Infection Prevention and Control

**Figure 7:**
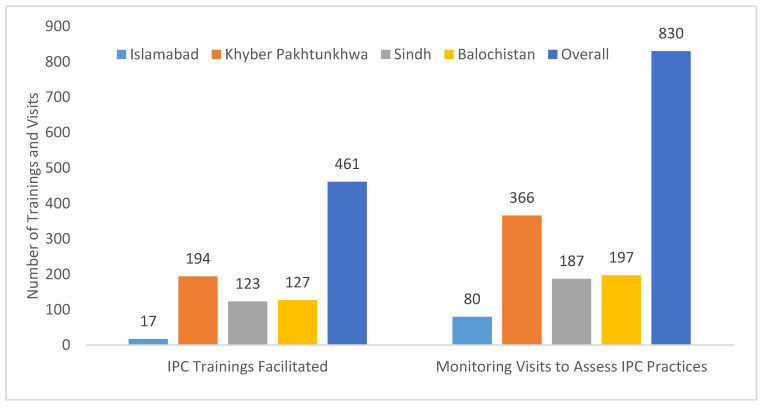
Infection Prevention and Control (IPC) training sessions facilitated and monitoring visits conducted by NSTOP officers to assess IPC practices during the COVID-19 response, Pakistan, March–July 2020.

Infection Prevention and Control (IPC) refresher training sessions were essential to keep frontline HCPs updated about measures for protecting themselves and others from COVID-19 infection. This required frequent visits to facilities to assess their IPC practices and identify areas where further training and focus were required. During these visits, NSTOP officers provided hands-on training on standard IPC practices to health professionals (doctors, nurses, paramedics, and janitor staff). Overall, NSTOP officers facilitated 531 IPC training sessions and conducted 1035 visits to health facilities to monitor for adherence to standard IPC practices (Table 1, Figure 7).

## 4. Discussion

The Pakistan NSTOP program, a group of field epidemiologists focused on the country’s efforts to eradicate polio, played a crucial role in the early phase of Pakistan’s COVID-19 response. During the first five months of Pakistan’s COVID-19 response from March to July 2020, NSTOP officers supported various aspects of the response, including coordination, detection and response activities, surveillance, quarantine/isolation management, training and orientation sessions for healthcare personnel, data analysis, community engagement, and risk communication. They successfully investigated 32,729 suspected COVID-19 cases, of which about one-third were confirmed cases, and facilitated the collection and dispatch of >57,000 samples from these cases. They also trained close to 10,000 healthcare personnel, handled more than 27,000 public inquiries, enlisted the support of >4000 religious leaders and community influencers, and established and managed >1700 quarantine/isolation stations. These contributions demonstrate the impact of having a well-trained, capable polio workforce in the context of an emergent public health crisis within a resource-limited setting.

The presence of the NSTOP program at the federal, provincial, divisional, and district levels greatly facilitated the ability of officers to support the country’s COVID-19 response at all levels. Taking advantage of the existing incident management structure of the polio program, with emergency operations centers and polio control rooms established at all administrative levels, officers were able to seamlessly transition into leadership and vital roles for the management of the COVID-19 response. The polio EOCs at the national and provincial levels, as well as divisional and district polio control rooms, were realigned to support the COVID-19 response. NSTOP officers served as leads and members of provincial and district command and control committees, and they led and supported rapid response teams. Collectively, they led or participated in over 2100 coordination committee meetings in addition to nearly 3800 evening review meetings. Taking advantage of the robust system in place for poliovirus surveillance in the country, they facilitated more than 400 COVID-19 surveillance training sessions and trained >1500 surveillance/screening teams. In short, they were the bedrock of the early and decisive phase of Pakistan’s COVID-19 response.

Modeled after the World Health Organization’s STOP program [10], the Pakistan NSTOP program continues to play a crucial role in Pakistan’s polio eradication efforts. The program was launched in response to the declaration of polio eradication as a public health emergency by the World Health Assembly in 2011 [11]. NSTOP officers are trained through Pakistan’s FELTP to support disease surveillance and outbreak response during routine situations as well as during emergency responses. Assigned to work in the most consequential districts and provinces for the polio program, NSTOP officers support the planning and implementation of SIAs, AFP surveillance, outbreak response, and strengthening routine immunization activities in line with the National Emergency Action Plan for polio eradication. Their public health and field epidemiology experience gleaned from their support for the polio program helped to hone their emergency preparedness skills and proved decisive in responding to the early phase of the country’s COVID-19 response. The NSTOP program was nimble in adapting its manpower resources and infrastructure towards the COVID-19 response once an outbreak was declared. Officers were quick to adapt to the challenges and demands of responding to an outbreak of a novel disease, translating their polio incident management skills to overcome hurdles with coordinating the COVID-19 response at a time when there was limited knowledge of the disease and testing capacity.

A similarly structured polio program in Nigeria, also known as the NSTOP program, made important contributions to the West African nation’s COVID-19 response [12]. The NSTOP program in Nigeria had previously been involved in case-based surveillance and management efforts in the country’s successful response to the Ebola outbreak that occurred in 2014 [13]. Whether in Pakistan or Nigeria, these programs underscore the value and impact of having a well-trained, locally adapted public health workforce to respond to and manage emergent health threats. Their familiarity with the communities they serve, by virtue of being recruited from and embedded within these communities, greatly facilitates their ability to lead in times of crisis [14]. Being integral to the national public health infrastructures of their countries enhances access and ensures they can work collaboratively down to the lowest administrative levels to tackle the most challenging public health issues [15]. This includes being able to access areas that are hard-to-reach or security-compromised [16,17].

The success of NSTOP officers in supporting the early phase of the COVID-19 response in Pakistan also serves as an exemplar of how the partnership between international organizations with national governments can help build up the human resource capacity of low- and middle-income countries, enabling them to rise to the challenge of meeting their own healthcare needs in times of crisis. The CDC, a leading partner in GPEI, provides substantial financial, logistical, and technical support for the NSTOP program. A major reason for the CDC’s support for the NSTOP program is to reduce the level of reliance on foreign experts who may not be culturally attuned to the environments where they work, recognizing that the most effective way to reach and engage communities with health interventions is through a well-trained and locally adapted workforce derived from within the communities themselves. Pakistan’s NSTOP officers demonstrated the value of this approach through their stellar contributions to the COVID-19 response, arguably the most consequential global health crisis in a century.

One significant limitation of this report is that it only spotlights the contributions of NSTOP officers to the early phase of the COVID-19 response in Pakistan prior to the introduction of vaccines against the disease. As a result, it does not highlight the role these officers could have played in implementing the rollout and safe delivery of COVID-19 vaccines in the country, given their extensive experience in planning and implementing polio vaccination activities on a large scale. The report does not analyze the impact of COVID-19 surveillance activities, based on case definition criteria, on the ability to detect and respond to cases. We also did not examine how the prevalence and mortality associated with COVID-19 could have varied based on deployments of NSTOP officers, a constraint necessitated by priorities in the early phase of the pandemic, when the unpredictable spread of the disease required prompt, pragmatic steps as opposed to methodical research activities to contain the outbreak. Despite these limitations, the report still provides valuable information on the impact of a highly trained polio workforce in responding to the COVID-19 pandemic.

## 5. Conclusions

This report details NSTOP contributions to the early phase of the COVID-19 response in Pakistan, demonstrating the value of a well-trained, locally adapted workforce in responding to a public health crisis. With the resurgence of polio in Pakistan [18], NSTOP officers will again be working in the most challenging areas of the country to eliminate poliovirus from the country. Achieving this will require the same determined, focused commitment with which they responded to the COVID-19 outbreak at its onset. In the long run, the NSTOP program in Pakistan could serve as a bridge towards strengthening the overall immunization infrastructure in the most resource-limited areas of the country, as the STOP program has achieved in countries like South Sudan [19]. This, along with the response capabilities already demonstrated during the COVID-19 pandemic, illustrates the value of polio investments beyond eradicating the disease to encompass having a workforce that is ready to respond to extant and emergent disease threats in any and all situations.

## Figures and Tables

**Table 1:** Summary of activities in support of the Pakistan COVID-19 response conducted by NSTOP officers in March–July 2020.

Key Indicators	District NSTOP Officers (n = 56)	TL/DTFs Officers (n = 8)	Overall (n = 64)	Average per Officer Contribution
A. Coordination: Involvement in response meetings				
DCCC/Task Team/RR meetings facilitated or attended	1717	387	2104	33
COVID-19 response-related ERMs or daily meetings facilitated	3358	425	3783	59
Security meetings attended	417	33	450	7
B. Case detection and Management				
COVID-19 suspected cases investigated	30,461	2268	32,729	511
COVID-19 confirmed cases investigated	9445	2058	11,503	180
COVID-19 suspected refusals identified and investigated	468	240	708	11
COVID-19 cases followed up for 14 days	11,381	2332	13,713	214
COVID-19 case contacts traced/identified	13,610	1673	15,283	239
COVID-19-positive cases transferred to isolation centers	2170	990	3160	49
Samples of suspected COVID-19 cases for which collection was facilitated	52,647	4546	57,193	894
Samples for which transportation was facilitated	50,549	4163	54,712	855
C. Risk Communication and Community Engagement				
Mass media programs (TV/radio) attended	136	98	234	4
Support as technical focal person to the PEOC Coordinator/Commissioner/DC/ADC in interactions with the media	1126	180	306	5
Community engagement sessions facilitated	1017	79	1096	17
Participants in community sessions	17,888	546	18,434	288
Calls at DPCR/Call Center supported as technical expert	25,178	2183	27,361	428
Facilitated the religious leaders’ engagement through DPCR	1843	54	1897	30
Facilitated the influencers’ engagement through DPCR	2114	197	2311	36
D. Training and Orientation Sessions				
COVID-19 response training sessions for healthcare providers facilitated	1100	104	1204	19
Participants in response training sessions	8801	1103	9904	155
Training sessions on PPE use facilitated	775	53	828	13
Participants in PPE use training sessions	4257	554	4811	75
Training sessions monitored	518	78	596	9
E. Surveillance and Screening				
COVID-19 surveillance committee meetings attended	681	65	746	12
COVID-19 surveillance training sessions facilitated or monitored	367	50	417	7
Number of surveillance/screening teams trained	1369	185	1554	24
F. Establishment and Maintenance of Quarantines/Isolations				
Quarantine/isolation centers for which establishment was supported	1681	79	1760	28
Visits to quarantine/isolation facilities	1251	180	1431	22
G. Infection Prevention and Control				
Training sessions on IPC facilitated	461	70	531	8
Facility visits to assess IPC practices conducted	830	205	1035	16

Abbreviations: DCCC = District Command and Control Committee, RRT = Rapid Response Team, ERM = Evening Review Meetings, TL = Team Lead, DTF = Divisional Task Force, PEOC = Provincial Emergency Operation Center, DC = Deputy Commissioner, ADC = Additional Deputy Commissioner, DPCR = District Polio Control Room, IPC = Infection Prevention Control, PPE = Personal Protective Equipment.
